# Strangulated obturator hernia: a case report with literature review

**DOI:** 10.11604/pamj.2019.32.144.14846

**Published:** 2019-03-26

**Authors:** Walid Mnari, Badii Hmida, Mezri Maatouk, Ahmed Zrig, Mondher Golli

**Affiliations:** 1 Imaging Department FB University Hospital Monastir, Tunisia

**Keywords:** Hernia, obturator foramen, bowel obstruction, CT scan

## Abstract

Obturator hernia is rare. It occurs when part of the pelvic contents protrude through the obturator foramen. It is a diagnostic challenge in the emergency department since the signs and symptoms are non-specific. It often occurs in elderly, emaciated and chronically ill women. The clinical picture include intestinal obstruction with abdominal pain, nausea and vomiting. The treatment is only surgical. Delayed diagnosis of this condition usually leads to a high mortality rate. We report the case of an 83-year-old woman with a strangulated obturator hernia. The hernia was discovered early by computed tomography and was treated by emergency laparotomy. We emphasize on the rule of CT scan to establishing a prompt preoperative diagnosis of an obturator hernia, appropriate planning of surgical intervention and thus optimizing the outcome.

## Introduction

Obturator hernia is a rare abdominal wall hernia with incidence of 1% [1]. It occur when the intestine protrudes through a defect in the obturator foramen and into the obturator canal [2]. It most commonly presents as non-specific signs of acute intestinal obstruction with contents being small bowel in majority of cases. Obturator hernias are potentially the most lethal of all abdominal wall hernias due to diagnosis delay and complications of this unknown pathology. Various imaging modalities have been applied to establish the diagnosis but the CT scan has superior sensitivity and accuracy [3]. Here we present a case of obturator hernia causing bowel obstruction which was diagnosed by CT scan and was successfully operated in emergency.

## Patient and observation

**Figure 1:**
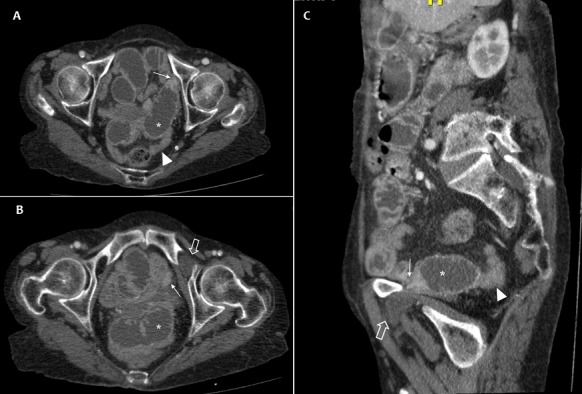
(A) axial pelvic CT scan; (B,C) multiplanar reconstructed images through the left obturator canal, showed a dilated loop of small bowel (asterisk) upstream of a strangulated point (arrow) caused by a left obturator hernia (large arrow). Noted the collapsed loops down and colon (arrowhead)

A frail 83-year-old woman was admitted with a 48 h history of acute abdominal pain, absolute constipation and vomiting. She had type 2 diabetes and had never undergone abdominal surgery. She reported two episodes of colicky hypogastric pain with lower abdominal swelling in the last few weeks that resolved spontaneously. There is no pain in the lower limbs especially in inner thigh or the knee. Clinical examination showed a moderate built patient with a temperature of 37.7°C, dry tongue and sunken eyes. On local examination, she had a distended abdomen with generalized tenderness and no groin hernias. Other systems were essentially normal. Rectal examination and bloods were unremarkable. Plain X-rays revealed dilated bowel loops with multiple central air-fluid levels suggestive of complete small bowel (SB) obstruction with no signs of perforation. Urgent CT scan of the abdomen and pelvis performed with contrast (Figure 1). It revealed a dilated SB loops with a transition point caused by a lateral pinching loop of SB with herniation through the left obturator foramen; the loops down and the colon were collapsed. There was a small effusion into the hernia sac with no other signs of severe bowel obstruction. The diagnosis of strangulated small-bowel obstruction secondary to a left obturator hernia was confirmed and the decision was to carry out surgery. At laparotomy, a strangulated centimeter segment of ileum was resected from the right obturator hernia defect and a side-to-side anastomosis was done. The obturator defect was closed with simple sutures. Patient was kept in high intensity care unit for 3 days. She was discharged after 10 days.

## Discussion

Obturator hernias are characterised by bowel herniating between the obturator and the pectineus muscles. They are a rare type of abdominal hernia. With the nickname “little old lady's hernia”, they are often seen in elderly, chronically ill and thin women [1]. The hernia sac can also contain appendix, Meckels diverticulum, omentum, ovary, fallopian tube and even uterus [4]. Due to non-specific signs and symptoms, the diagnosis and treatment of this condition are usually delayed, which has led to high mortality rate and can be very difficult to diagnose clinically [5]. The obturator foramen of iliac bone is sealed by a thick membrane which drilled by the obturator canal. This 2-3cm tunnel contains the obturator neurovascular bundle. It is through this deficiency that an obturator hernia occurs facilitated by the atrophy and loss of preperitoneal fat around obturator vessels. Multiparity, constipation, ascites, and causes of raised intra-abdominal pressure are the other predisposing factors. These hernias are more common on the right due to the left position of sigmoid colon in the pelvis [6]. The most common clinical presentation of obturator hernia is intestinal obstruction, with nausea, vomiting, abdominal pain, distension and weight loss, but this is nonspecific [7]. The hernia orifice is small, so bowel pinching and strangulation are frequent and mortality remains high (25%-47.6%) [7].

Howship-rhomberg sign is pain radiating down the medial aspect of the thigh to the knee and less often to the hip caused by compression and irritation of the obturator nerve within the canal, this pathognomonic sign is seen in 15-20% of patients. Because symptoms are nonspecific and determined physical findings are often absent, diagnosis of obturator hernia usually delayed until laparotomy performed for treatment of bowel obstruction or peritonitis. Various imaging tools have been formerly used to establish the diagnosis, including barium enema fluoroscopy, ultrasonography and herniography. Actually, emergency multidetector CT scanning could lead to rapid diagnosis and early surgical intervention and thus optimizing the outcome. The use of CT scan in detecting obturator hernia was first reported by Meziane .*et al.* in 1983 [8]. The common CT scan finding is herniated loop of distal small bowel extending through the obturator foramen between the pectineus and obturator externus muscles. In incarcerated hernia, CT scan showed associated bowel loop dilatation in the abdomen and in severe cases, the bowel becomes edematous ischemic, leading to gangrene and perforation. Since the use of CT scan, pre-operative diagnosis rate was improved from 43% to 90% in some reports [2]. The only treatment for obturator hernia is surgery [1, 2]. In the emergency setting, the abdominal approach via a low midline incision is favored. Resection of the involved portion of bowel is sometimes required because of gangrenous change or perforation.

## Conclusion

An obturator hernia is very rare and difficult to diagnose. It occurs when part of the pelvic contents protrude through the obturator foramen. In elderly and chronically ill women with signs of bowel obstruction, this type of hernia must be considered. Abdominal CT scan is actually the best diagnosis tool.

## Competing interests

The authors declare no competing interests.
